# Complete Genome Sequence of *Salinarchaeum* sp. Strain HArcht-Bsk1^T^, Isolated from Hypersaline Lake Baskunchak, Russia

**DOI:** 10.1128/genomeA.00505-13

**Published:** 2013-07-18

**Authors:** I. N. Dominova, D. Y. Sorokin, I. V. Kublanov, M. V. Patrushev, S. V. Toshchakov

**Affiliations:** Department of Genomic and Proteomic Research, Immanuel Kant Baltic Federal University, Kaliningrad, Russiaa; Winogradsky Institute of Microbiology, Russian Academy of Sciences, Moscow, Russiab; Department of Biotechnology, Delft University of Technology, Delft, The Netherlandsc

## Abstract

The complete genome sequence of a novel halophilic archaeon, *Salinarchaeum* sp. strain HArcht-Bsk1^T^, was determined using next-generation sequencing. The genome comprises a 3,255,260-bp circular chromosome with a G+C content of 66.7%. Automatic annotation of the genome revealed a single rRNA operon, 45 tRNAs, and 3,013 protein-coding gene sequences.

## GENOME ANNOUNCEMENT

Hypersaline inland lakes harbor a large and still not thoroughly tapped diversity of extremely halophilic *Euryarchaeota* growing optimally at salt-saturating conditions. Strain HArcht-Bsk1^T^ was isolated from the top aerobic sediment layer of the hypersaline chloride-sulfate Lake Baskunchak (south Russia). Cells of strain HArcht-Bsk1^T^ were occasionally motile pleomorphic flat rods during the exponential phase of growth, becoming irregularly shaped coccoids and discoids at the later stages. Strain HArcht-Bsk1^T^ grew optimally at 40°С (рН 7.5) and 3.5 M of NaCl. It grew organotrophically using various sugars as substrates. A BLAST search (1) of the HArcht-Bsk1^T^ 16S rRNA gene placed the novel isolate into the euryarchaeal nonvalid genus “*Salinarchaeum*.” “*S. laminariae*” strain R26^T^ (2) is its closest relative, with 98.5% sequence similarity. A BLAST search using the EzTaxon-e server (3) against 16S rRNA genes of valid species revealed that among the valid organisms the most similar sequence belonged to *Halovivax asiaticus* strain EJ-46^T^ (4), with 90.9% sequence similarity.

For determination of the complete genome sequence of *Salinarchaeum* sp. strain HArcht-Bsk1^T^, a combination of fragment (Nextera library with an average insert length of 500 bp) and long mate pair (PGM library with an average insert length of 2,200 bp) approaches were used. The fragment library was sequenced with two bar-coded runs of the Illumina MiSeq system, resulting in 5,919,667 101-bp and 222,683 250-bp paired-end reads. Reads were corrected with the Quake sequencing error correction tool (5). Overlapping pairs were merged with the paired-read merging tool of the CLC Genomics Workbench (parameters: mismatch cost, 4; gap cost, 5; maximum unaligned bases, 0; and minimum score, 10), and then all the reads were subjected to stringent quality trimming (error probability, 0.01; maximum number of ambiguities per read, 2). The mate-paired library was sequenced with the Life Technologies PGM System using a 314 chip. A total of 541,223 single PGM reads were imported in CLC Genomics Workbench (internal adapter clipping and splitting of reads into pairs were parts of the importing procedure). Single reads were excluded from the analysis and paired reads were trimmed according to the parameters described above. Finally, about 6 million paired-end and 449,031 high-quality mate-paired reads were obtained. Reads were assembled with CLC Assembler using recommended parameters (word size, 64; bubble size, 500) (6). Assembly resulted in one large contig, which was circularized using analysis of broken read pairs at sequence ends. After circularization, all the reads were mapped back to the contig and the mapping was examined manually to reveal potential misassemblies. Finally, a consensus genomic sequence was extracted and used for submission to GenBank and annotation.

The *Salinarchaeum* sp. genome comprises a 3,255,260-bp circular chromosome with a G+C content of 66.7%. Annotation of the genome was carried out with the NCBI Prokaryotic Genome Annotation Pipeline (http://www.ncbi.nlm.nih.gov/genome/annotation_prok/). The chromosome contains a single rRNA operon, 45 tRNA genes for all 20 standard amino acids, and 3,013 protein-coding sequences

### Nucleotide sequence accession number.

The genome sequence of *Salinarchaeum* sp. strain HArcht-Bsk1^T^ has been deposited in NCBI GenBank under the accession number CP005962.
